# Honokiol inhibits c-Met-HO-1 tumor-promoting pathway and its cross-talk with calcineurin inhibitor-mediated renal cancer growth

**DOI:** 10.1038/s41598-017-05455-1

**Published:** 2017-07-19

**Authors:** Murugabaskar Balan, Samik Chakraborty, Evelyn Flynn, David Zurakowski, Soumitro Pal

**Affiliations:** 1 Division of Nephrology, Boston Children’s Hospital, Boston, MA 02115 United States; 2 Department of Anesthesia, Boston Children’s Hospital, Boston, MA 02115 United States; 3 Harvard Medical School, Boston, MA 02115 United States

## Abstract

Honokiol (HNK) is a small molecule with potent anti-inflammatory and anti-tumorigenic properties; yet the molecular targets of HNK are not well studied. Hyperactivation of the receptor tyrosine kinase c-Met and overexpression of the cytoprotective enzyme heme oxygenase-1 (HO-1) play a critical role in the growth and progression of renal cell carcinoma (RCC). Interestingly, the calcineurin inhibitor (CNI) cyclosporine A (CsA), an immunosuppressant used to prevent allograft rejection, can also increase the risk of RCC in transplant patients. We studied the potential role of c-Met signaling axis on CNI-induced renal tumor growth and tested the anti-tumor efficacy of HNK. Importantly, CNI treatment promoted c-Met induction and enhanced c-Met-induced Ras activation. We found that HNK treatment effectively down-regulated both c-Met phosphorylation and Ras activation in renal cancer cells. It inhibited the expression of both c-Met- and CNI-induced HO-1, and promoted cancer cell apoptosis. *In vivo*, HNK markedly inhibited CNI-induced renal tumor growth; and it decreased the expression of phospho-c-Met and HO-1 and reduced blood vessel density in tumor tissues. Our results suggest a novel mechanism(s) by which HNK exerts its anti-tumor activity through the inhibition of c-Met-Ras-HO-1 axis; and it can have significant therapeutic potential to prevent post-transplantation cancer in immunosuppressed patients.

## Introduction

Honokiol (HNK) is a natural bioactive compound originally derived from the seed and bark extract of magnolia plant species (Magnolia obovata), which has been used in traditional Chinese and Japanese medicine^1^. Chemically, HNK is characterized as a small bi-phenolic lignan^1, 2^. Recent reports have demonstrated the anti-inflammatory, anti-angiogenic, and anti-tumor properties of HNK^1^. It has been demonstrated that the treatment with HNK can inhibit receptor tyrosine kinase (RTK)-mediated events and induce pro-apoptotic signaling pathway(s) in tumor cells^3^. Thus, HNK can be a very promising agent to prevent or restrict tumor growth.

The RTK c-Met and its ligand HGF play a significant role in the growth and progression of renal cancer^4, 5^. c-Met is overexpressed in both clear cell and papillary renal cell carcinoma (RCC)^6, 7^. Notably, both HGF and c-Met are located on chromosome 7 and is often amplified in clear cell RCC; and germline c-Met mutations in papillary renal cancer promotes constitutive activation of c-Met^8, 9^. Homodimerization of c-Met upon binding of HGF induces phosphorylation of two tyrosine residues (Y1234 and Y1235) present within the catalytic loop of the tyrosine kinase domain^10^. Subsequently, phosphorylation of tyrosines 1349 and 1356 in the C-terminal tail recruits signaling adaptor proteins GRb2, SHC and CRK, which mediate the activation of multiple signaling pathways^11^. c-Met activation promotes the expression of anti-apoptotic molecules in renal cancer cells^4^. However, the effect(s) of HNK on c-Met-mediated pro-tumorigenic pathway in renal cancer cells, including tumor angiogenesis, is completely unexplored.

We have recently demonstrated that the induction of c-Met promotes over-expression of the cytoprotective molecule heme oxygenase-1 (HO-1) for the survival of renal cancer cells^4^. HO-1 is a catalytic enzyme involved in the degradation of potent inflammatory agent heme into carbon monoxide (CO), billiverdin and ferrous iron^12^. Although it plays a beneficial role in repairing tissue injury, cancer cells over-express HO-1^13, 14^. By the virtue of its cyto-protective effects, HO-1 plays an important role in tumor growth and the prevention of chemotherapeutic drug-induced apoptosis^15^. HO-1 has also been shown to induce tumor angiogenesis^16^. Many transcription factors, such as Nrf2 and Bach1 that regulate HO-1 expression, also play a major role in tumor progression^4, 17, 18^. However, the effect of HNK in modulating c-Met-induced HO-1 expression in RCC has not been studied.

Although aberrant c-Met and other RTK hyper-activation are the major drivers of renal cancer growth, recent reports clearly suggest an alarmingly high incidence of renal cancer in patients receiving calcineurin inhibitors (CNIs) as immunosuppressive therapy for the treatment of immune disorders or after having organ transplants^19–21^. The CNI Cyclosporine A (CsA) inhibits T cell activation, by down-regulating the phosphatase activity of calcineurin and preventing the nuclear translocation of NFAT, a crucial factor for T cell activation, and thereby suppresses immune system^22^. However, independent of its immunosuppressive functions, which may lead to immune escape of cancer cells, the CNI treatment can directly induce tumor-promoting pathways^23–26^. We have shown that the CNI treatment promotes Ras activation and induces the expression of cytoprotective molecule HO-1 for the rapid growth and survival of renal cancer cells^24, 27^. We have demonstrated that c-Met is also a potent inducer of the Ras-HO-1 pathway^4^. Interestingly, in our initial studies, we have found that HNK can inhibit CNI-induced Ras activation and HO-1 over-expression in renal cancer cells^27^. However, we have not yet investigated the effect of HNK treatment on c-Met-induced tumorigenic pathway(s) and it’s possible cross-talk with CNI-induced events; and if HNK can restrict renal tumor growth *in vivo*.

In this study, we report that HNK treatment significantly inhibits c-Met phosphorylation and c-Met-/CNI-induced Ras activation and HO-1-overexpression in renal cancer cells; and it restricts CNI-induced renal tumor growth *in vivo*. Our observations suggest that HNK can act as a novel therapeutic agent for c-Met-Ras-HO-1-mediated renal cancer, with particular importance to tumor growth in immunosuppressed patients. In future, we suggest that along with anti-tumorigenic function, the anti-inflammatory property of HNK can also be utilized to sustain the immunosuppression of transplant and other patients with immune disorders.

## Results

### HNK treatment down-regulates HGF-induced c-Met phosphorylation, Ras activation and HO-1 over-expression, and its cross-talk with CNI-induced tumor-promoting pathway(s) in renal cancer cells

**Figure 1:**
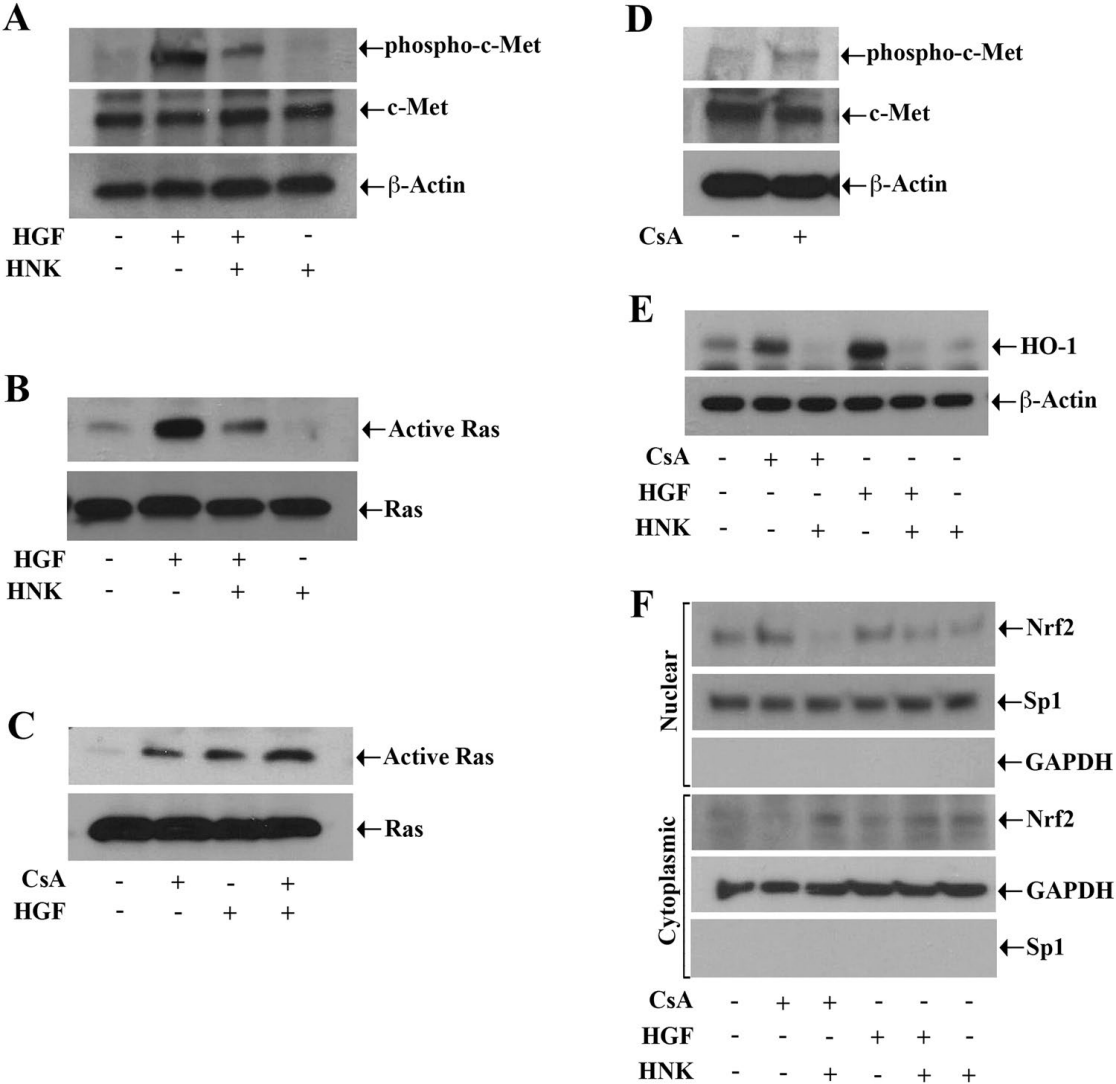
HNK treatment down-regulates HGF-induced c-Met phosphorylation; and it inhibits both HGF- and CNI-mediated Ras activation and HO-1 over-expression. (**A**) 786-O cells were pre-treated with either HNK (20 μM)/vehicle alone for 2 h, and then treated with either HGF (50 ng/ml) or vehicle alone for 30 min. Following treatment, the cell lysates were used to measure the levels of phospho-c-Met, c-Met, and β-actin (internal control) by Western blot analysis. (**B**) 786-O cells were treated as described in (**A**). Following treatment, cell lysates were prepared utilizing a Ras activation kit as described under “Materials and Methods” section, and the expression of GTP-bound Ras was subsequently analyzed by Western blot. (**C**). 786-O cells were pre-treated with either CsA (5 μM)/vehicle alone for 2 h, and then treated with either HGF (50 ng/ml) or vehicle alone for 30 min. Following treatment, the expression of GTP-bound Ras was analyzed by Western blot. (**D**) 786-O cells were treated with either CsA (5 μM) or vehicle alone for 30 min and cell lysates were utilized to measure the levels of phospho-c-Met, c-Met, and β-actin by Western blot analysis. (**E**) 786-O cells were pre-treated with either HNK (20 μM) or vehicle alone for 2 h and then treated with different combinations of HGF (50 ng/ml), CsA (5 μM) or vehicle alone for 24 h. Following treatment, the cell lysates were used to measure the expression of HO-1 and β-actin. F. 786-0 cells were treated as described in (**E**), and following treatment nuclear and cytoplasmic fractions were isolated from cells, and Western blot analysis was performed to measure the expression of Nrf-2 in each of the fractions. The purities of nuclear and cytoplasmic fractions were evaluated by the expression of SP-1 and GAPDH respectively. (**A** to **F**) are representative of three independent experiments. For all Western blots (**A**–**F**), the bands from adjacent lanes were cropped from the same blot (full-length blots are included in a supplementary file).

In our previous reports we have demonstrated that both c-Met phosphorylation and Ras activation play major roles in orchestrating growth-promoting signals in renal cancer cells^4^. Here, we checked whether HNK treatment has any effect on c-Met-mediated signaling and Ras activation in 786-O and ACHN renal cancer cells. We observed that the c-Met ligand HGF significantly induced c-Met phosphorylation, which was markedly reduced following HNK treatment in both 786-O and ACHN (data not shown) cells (Fig. 1A). We also found that HNK significantly inhibited the expression of basal as well as HGF-induced GTP-bound active Ras in renal cancer cells compared with vehicle-treated control (Fig. 1B).

Previously, we described that the treatment with CNI (CsA) can induce Ras activation to promote the growth and progression of renal cancer; however, the role of CsA in conjunction with c-Met signaling axis has not been studied^24^. Here, we found that in the presence of CsA treatment, HGF-induced Ras activation was further increased (Fig. 1C); and we also noted that CsA moderately induced the basal c-Met phosphorylation in renal cancer cells compared with vehicle-treated control (Fig. 1D).

As discussed earlier, the expression of HO-1 plays an important role in both c-Met and CsA-mediated tumorigenic pathways in renal cancer cells. Here, we examined the effect of HNK on c-Met and CsA-induced HO-1 expression. We observed that HNK treatment significantly inhibited both HGF and CsA-induced HO-1 expression (Fig. 1E). It is established that the transcription factors Bach-1 and Nrf2 regulate HO-1 transcription in a negative and positive manner, respectively. Importantly, the nuclear translocation of Nrf2 has been implied as one of the critical events in HO-1-mediated growth and survival of cancer cells^4^. Here, we wished to study the effect of HNK on Nrf2 nuclear translocation. Analyzing the nuclear versus cytoplasmic localization of Nrf2, we found that HNK treatment markedly inhibited both c-Met and CsA-induced Nrf2 nuclear translocation (Fig. 1F, *top three panels*); conversely, we also found that HNK treatment retained Nrf2 in the cytoplasmic compartment, as seen with increased Nrf2 expression in the cytoplasmic fraction (Fig. 1F, *lower three panels*). Together, our results demonstrate that HNK significantly down-regulates c-Met- and CNI-induced Ras activation and Nrf2-mediated HO-1 over-expression in renal cancer cells.

### HNK inhibits c-Met-induced growth and survival of renal cancer cells and promotes apoptosis

**Figure 2:**
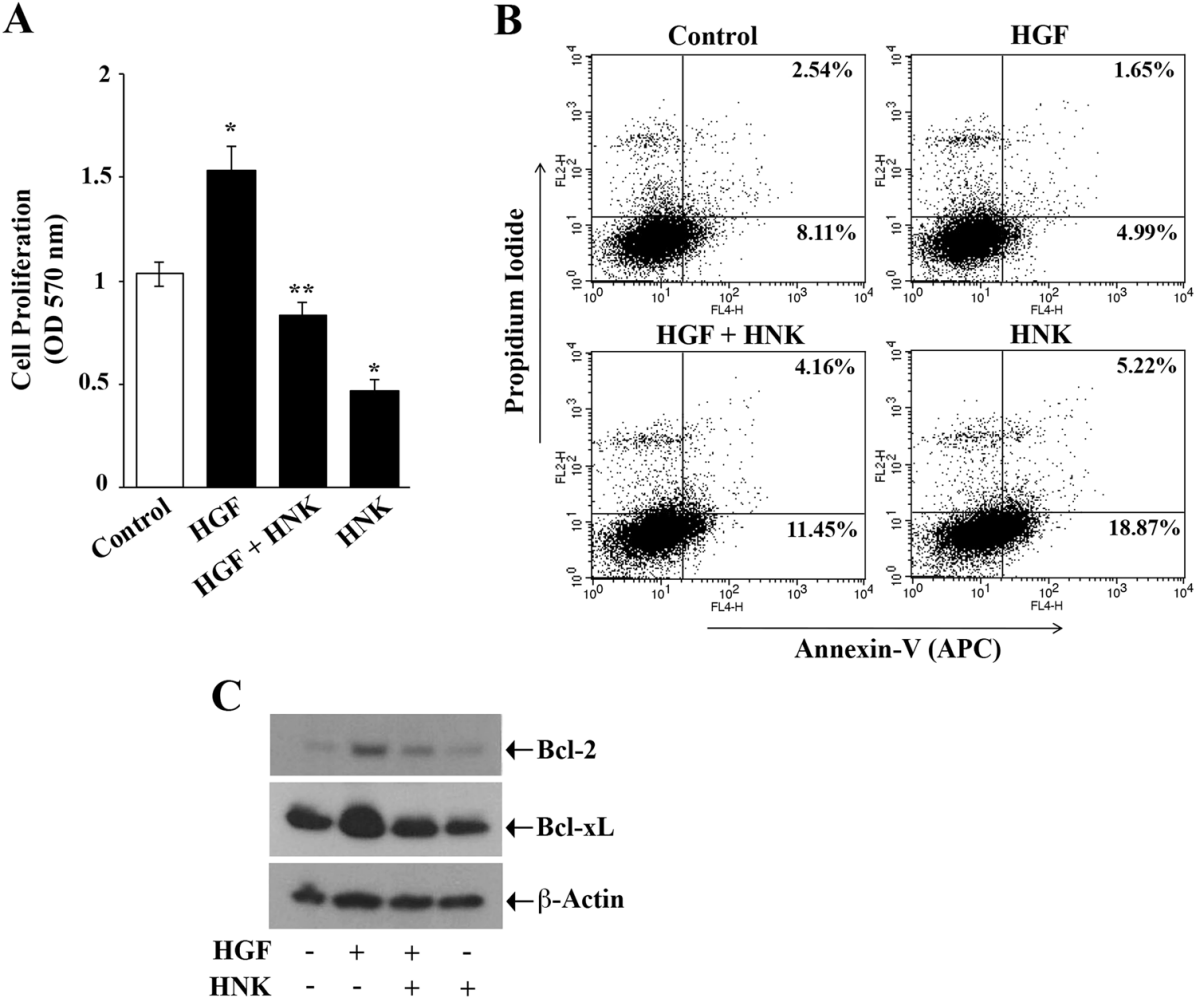
HNK inhibits c-Met-induced growth and survival of renal cancer cells and promotes apoptosis. (**A**) 786-O cells were pre-treated with either HNK (20 μM)/vehicle alone for 2 h and then treated with either HGF (50 ng/ml) or vehicle alone for 24 h. Following treatment, cell proliferation was measured by MTT assay. (**B**) 786-O cells were treated as described in (**A**), and following treatment, apoptotic index of the cells was determined by annexin V (APC) and propidium iodide staining. (**C**) 786-O cells were treated as described in (**A**). Following treatment, the cell lysates were used to measure the expression of Bcl-2, Bcl-xL and β-actin. (**A**) The columns represent the mean ± S.D. of triplicate readings of two different samples. (**B**,**C**) Are representative of three independent experiments. *p < 0.05 compared with vehicle treated control and **P < 0.05 compared with only HGF-treated cells. In 2 C, the bands from adjacent lanes were cropped from the same blot (full-length blots are included in a supplementary file).

We have previously reported that c-Met plays a critical role in protecting renal cancer cells from apoptosis^4^. Here, we wished to study the effect of HNK in counteracting c-Met-induced cell survival. By MTT assay, we observed that HNK treatment significantly inhibited HGF/c-Met-induced proliferation of renal cancer cells (Fig. 2A). We also examined the effect of HNK on c-Met-mediated protection of renal cancer cells from apoptosis. Through annexin V and propidium iodide staining, and by analyzing the cellular apoptotic index through flow cytometry, we observed that HGF treatment protected renal cancer cells from apoptosis, whereas HNK significantly increased cell death in both HGF-treated and control cells (Fig. 2B). When compared to vehicle-treated control, the percentage of total apoptotic cells (early + late) decreased from (8.11% + 2.54%) = 10.65% to (4.99% + 1.65%) = 6.64% following HGF treatment; HNK down-regulated this protective effect of HGF and increased apoptosis from 6.64% to 15.61% (11.45% + 4.16%). HNK treatment also markedly increased the basal apoptosis from 10.65% to 24.09% (18.87% + 5.22%) compared with control cells.

We have previously reported that c-Met-mediated expression of anti-apoptotic molecules such as Bcl2 and Bcl-xL plays major role in the growth and survival of renal cancer cells^4^. Here, we checked the effect of HNK on the expression of c-Met-induced Bcl2 and Bcl-xL expression. We found that HNK treatment markedly inhibited HGF-induced Bcl-2 and Bcl-xL expression as observed by Western blot (Fig. 2C). Together, we demonstrate that HNK down-regulated c-Met-induced survival of renal cancer cells through the modulation of cell proliferation and apoptosis.

### HNK down-regulates both c-Met- and CNI-induced migration of renal cancer cells, and tube formation of endothelial cells *in vitro*

**Figure 3:**
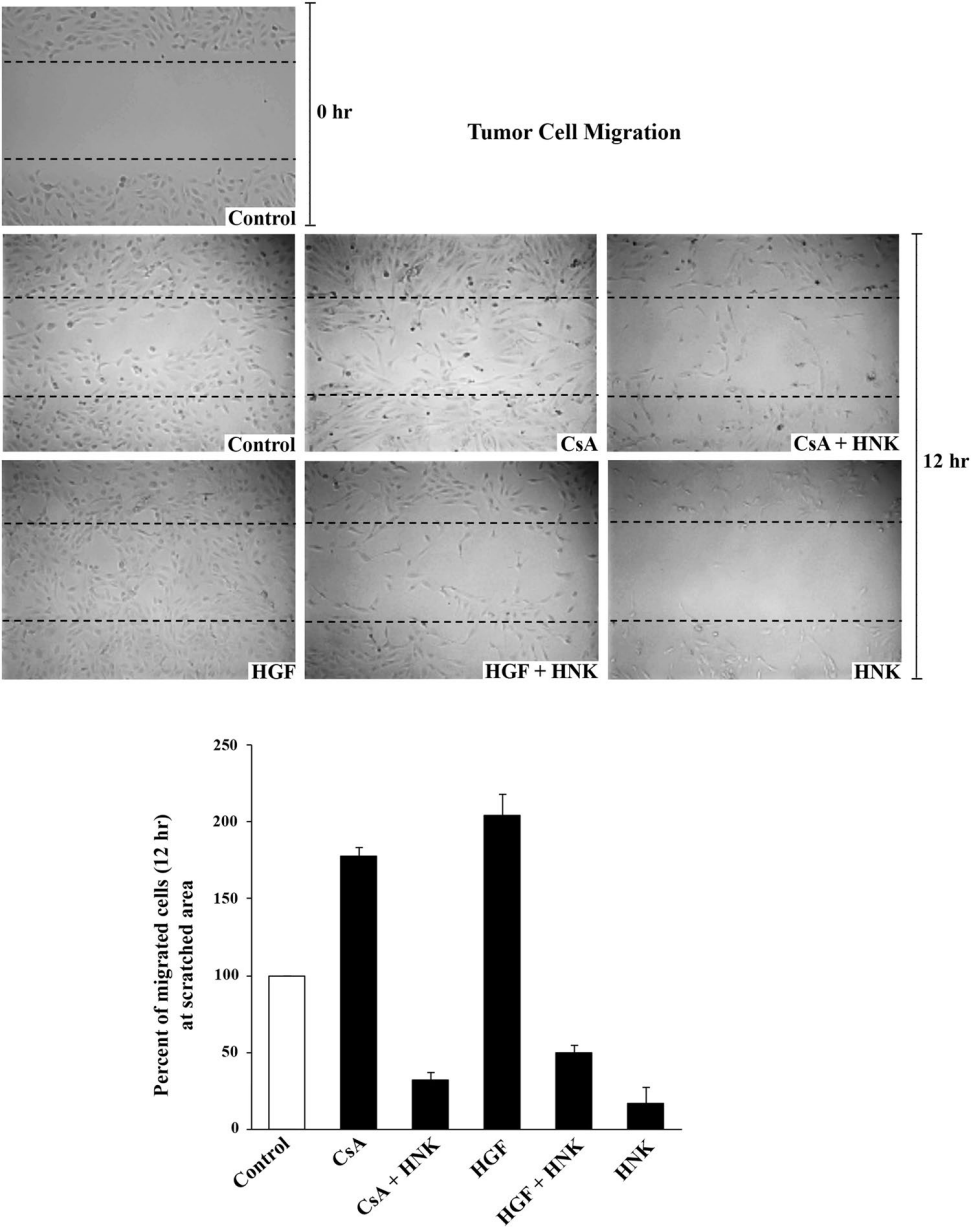
HNK down-regulates both c-Met- and CNI-induced migration of renal cancer cells: 786-O cells were pre-treated with either HNK (20 μM)/vehicle alone for 2 h and then treated with different combinations of HGF (50 ng/ml), CsA (5 μM) or vehicle alone. Following treatment, a wound healing assay (as described in the “Materials and Methods” section) was performed to assess the migration of cells. Images were taken immediately after scratching the cultures at 0 h (only control is shown) and 12 h later (shown for all treatments) using phase contrast microscope (photomicrographs, *top panel*). The number of cells migrated into the scratched wound area was quantified using NIH ImageJ software (*bottom* bar diagram). Images are representative of three independent experiments; and in bar diagram, the columns represent the mean ± S.D. of three independent measurements along the wound scratch.

Cancer cell migration plays an important role in tumor spread and metastasis^28, 29^. c-Met activation has been shown to contribute to metastasis of renal cancer cells; and CNI (CsA)-mediated signaling also confers tumor invasiveness by a direct effect on renal tumor cells and promotes metastasis^5, 30^. Since, in this study, we also found that CsA treatment potentiates c-Met-mediated signaling, we wished to study the effect of HNK on both c-Met and CsA-induced migration of renal cancer cells *in vitro* by performing a wound healing assay. The ability of cells to migrate and fill the wound was quantified at 0 h and 12 h. We observed that both HGF- and CsA-induced signals increased the number of migrated cells that filled the wound area compared with control cells; whereas HNK significantly inhibited the increased migration of cancer cells following treatment with both HGF and CsA (Fig. 3).

**Figure 4:**
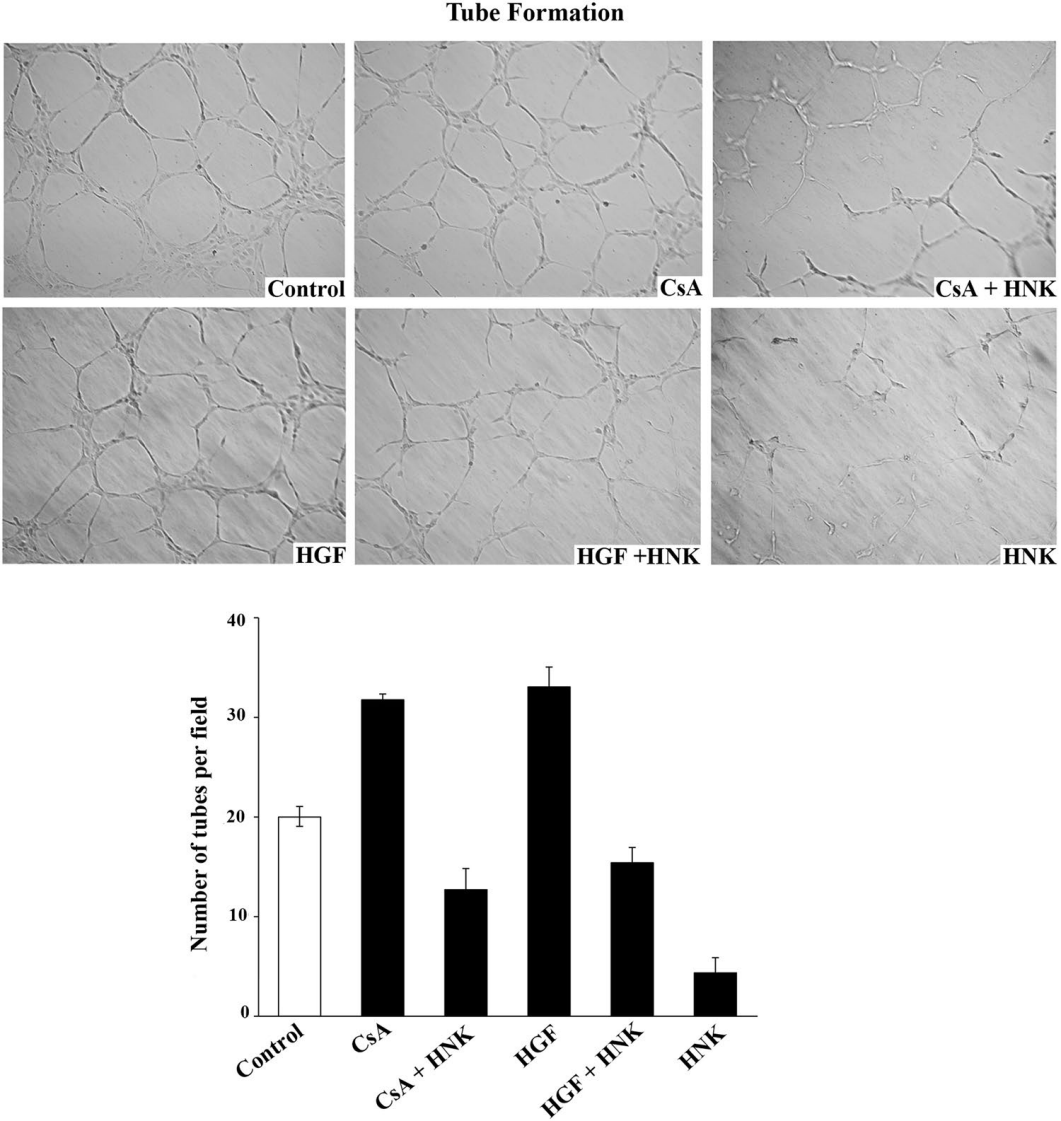
HNK down-regulates both c-Met- and CNI-induced secretion of angiogenic factors from renal cancer cells: The magnitude of secretion of angiogenic factors from renal cancer cells was assessed by *in vitro* tube formation assay using HUVEC cells (as described in “Materials and Methods” section). 786-O cells were pre-treated with either HNK (20 μM) or vehicle alone for 2 h and then treated with different combinations of HGF (50 ng/ml), CsA (5 μM) or vehicle alone for 12 h. Following treatment, cell culture medium was replaced with serum free culture medium and cells were grown for an additional 24 h. Subsequently, culture supernatants from each of the indicated treatments were collected and utilized to culture HUVEC cells on growth factor-reduced extracellular matrix (Geltrex). Representative images of capillary-like tube formation of HUVEC cells, taken with phase contrast microscope, are shown. The extent of tube formation was quantified by counting the number of uninhibited polygonal tube-like structures in five randomly selected microscopic fields (*bottom* bar diagram). Images are representative of three independent experiments; and in bar diagram columns represent the ±S.D. of three independent experiments.

Renal tumors are highly angiogenic, and both c-Met induction and CsA treatment can promote tumor angiogenesis^31–33^. Here, we examined how the CsA- and HGF-treated renal cancer cells can increase the ability of endothelial cells (HUVEC) to form tube-like structures (tube formation assay), an important step in the cascade of events that leads to new vessel formation; and if HNK can block the process. We observed that the supernatants from both HGF- or CsA-treated cancer cells increased the number of endothelial tubes formed compared with control; whereas the supernatants from cells grown in the presence of HNK significantly inhibited the tube formation ability of HUVEC cells (Fig. 4). Together, our observations suggest that HNK can effectively down-regulate c-Met- and CNI-induced renal cancer cell migration and endothelial tube formation.

### HNK treatment inhibits CNI-induced renal tumor growth *in vivo*; and it is associated with decreased tumor vasculature and increased tumor cell apoptosis

**Figure 5:**
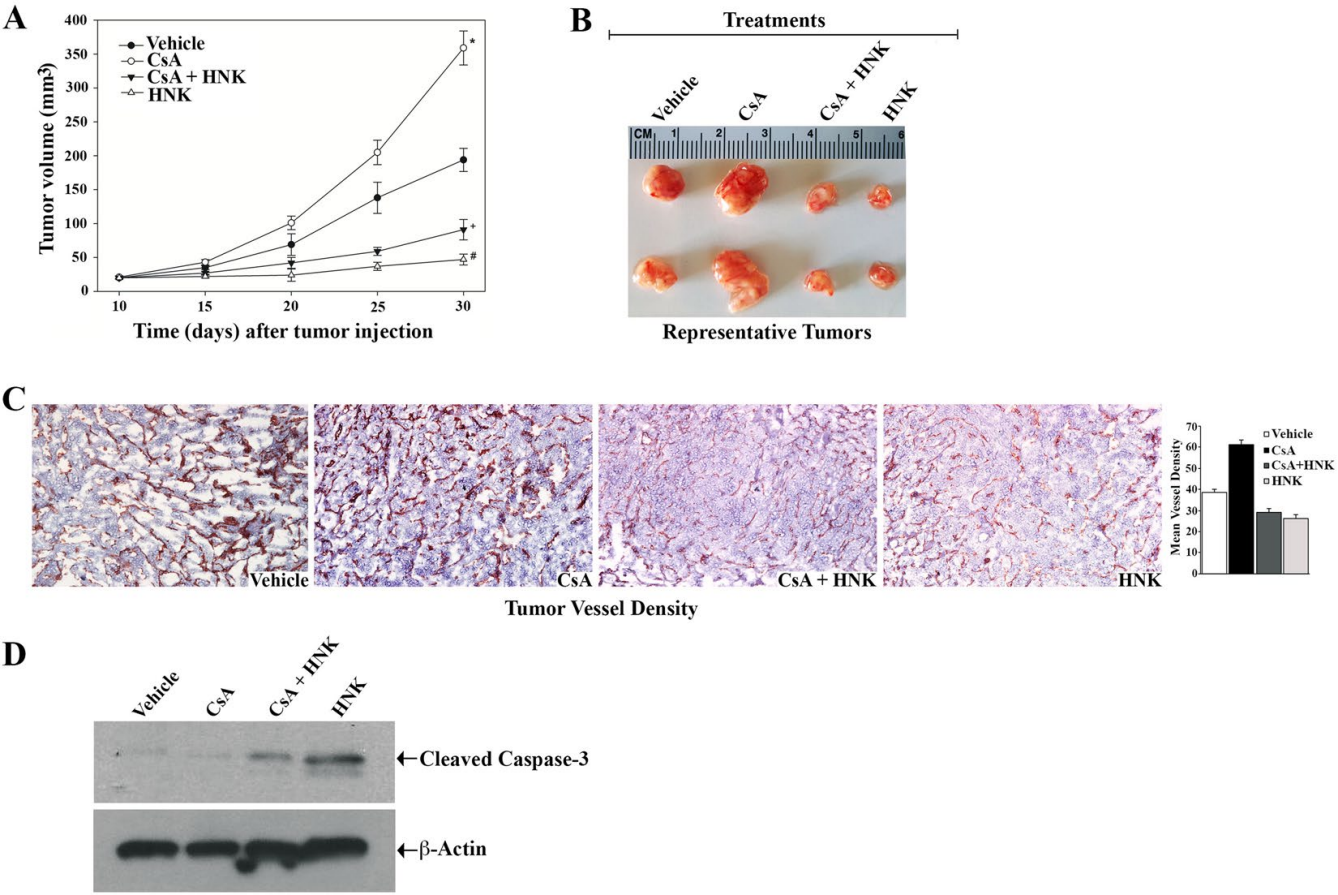
HNK treatment inhibits CNI-induced renal tumor growth *in vivo*; and it is associated with decreased tumor vasculature and increased tumor cell apoptosis. Nude mice were subcutaneously, xenografted with 786-O cells (1 × 10^6^), and when palpable tumor appeared around 10 days later, mice (n = 5 each group) were treated intraperitonealy with different combinations of CsA (10 mg/Kg/day) and HNK (2 mg/Kg/day); and the mice in control group were treated with vehicle alone. Treatments were continued for up to 20 days (i.e. 30 days after tumor injection). (**A**) Tumor volumes were calculated (as described in “Materials and Methods” section) for the four treatment groups. The mean change in tumor volume is shown in the tumor growth curve, (*CsA compared to vehicle treatment, p < 0.0001; ^+^(CsA + HNK) treatment compared to CsA alone, p < 0.0001; and #, HNK alone compared to (CsA + HNK) treatment, p < 0.0001). The data reflect representative of two independent experiments. (**B**) Tumors were excised after euthanizing mice at the end of study, and two representative tumors from each group is shown to depict tumor size. (**C**) IHC stains showing the expression of CD31 in harvested tumor tissues of mice from all treatment groups. Representative photomicrographs (magnification, x400) of tissue sections from each treatment group are shown. *Side panel*, bar diagram depicts mean vessel density calculated by standard grid counting of CD31-positive vessels. The data reflect ±S.D. of three different tissues from each group, in which three to four non-overlapping fields of each specimen were analyzed in a blinded manner. (**D**) Total tissue lysates, prepared from the excised tumors, were utilized to measure the expression level of cleaved caspase-3 and β-actin by Western blot. The bands from adjacent lanes were cropped from the same blot (full-length blots are included in a supplementary file). Representative blots of three different tumor tissue lysates from each group are shown.

We examined the effect of HNK on the growth of CNI (CsA)-induced renal tumor *in vivo* using a tumor xenograft model. 786-O cells were injected subcutaneously into the flank of nude mice. Once palpable tumors were formed, mice were treated with different combinations of established doses of CsA and HNK; and the vehicle-treated group served as control. As shown in Fig. 5A, there was a significant increase in tumor volume in CsA-treated mice compared with the vehicle-treated group. HNK treatment significantly inhibited tumor growth in CsA-treated as well as in vehicle-treated mice. The representative images depicting tumor size from different treatment groups at the end of the study is also shown (Fig. 5B).

Next, we examined the tumor vessel densities through CD31 staining. As expected, CsA treatment markedly increased vessel density compared with the vehicle-treated group. HNK treatment reduced tumor vessel density in both CsA- and vehicle-treated mice (Fig. 5C). To check the apoptotic status, we checked the expression of cleaved caspase-3 in the tumor tissues. We found that HNK increased the level of cleaved caspase-3 in both CsA- and vehicle-treated groups (Fig. 5D). Thus, our results suggest a potent anti-tumor function of HNK against CsA-induced renal tumor growth *in vivo*; and HNK inhibits CsA-induced increased tumor vessel density, and it promotes apoptosis of tumor cells.

### HNK down-regulates CNI-induced c-Met phosphorylation and HO-1 over-expression in renal tumors

**Figure 6:**
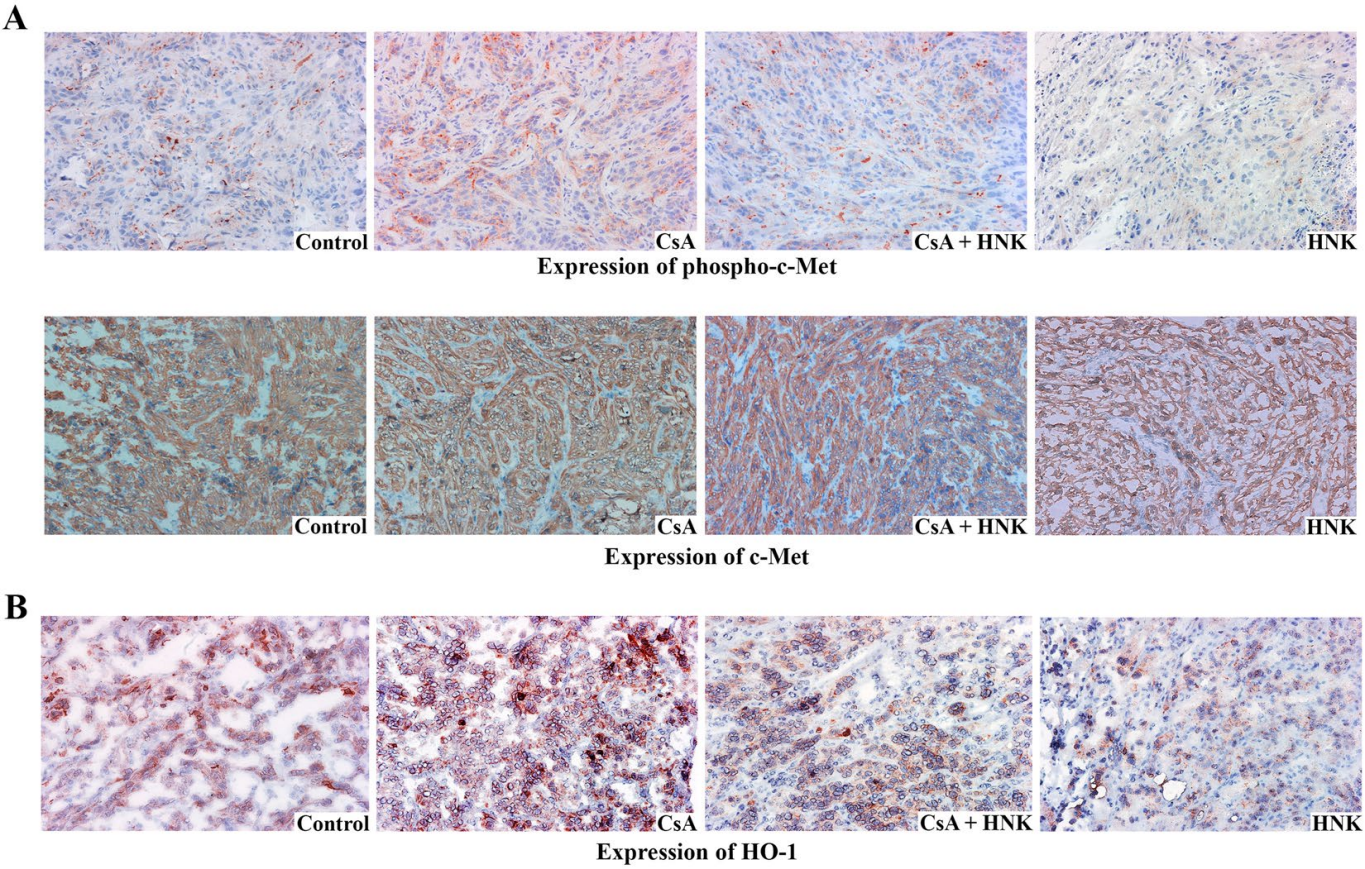
HNK treatment down-regulates CNI-induced c-Met phosphorylation and HO-1 over-expression in renal tumors. IHC stains showing the expression of phospho-c-Met (**A**, *top panel*) and c-Met (**A**, *bottom panel*) and HO-1 (**B**) in harvested tumor tissues (as described in Fig. 5). Representative photomicrographs (magnification x400) of three different tumor tissues from each group are shown.

We wished to examine the status of c-Met activation in tumor tissues obtained from CsA- and HNK-treated mice as described in the previous section. Through immunohistochemistry (IHC) (Fig. 6A), we observed that CsA treatment increased phospho-c-Met expression (red stain) compared with vehicle-treated control; and HNK markedly inhibited CsA-induced c-Met activation. However, there was no significant change in the expression of total c-Met in the tumors of the experimental groups. Corroborating with our *in vitro* data, CsA treatment also increased HO-1 expression (red stain) *in vivo*, and HNK treatment significantly inhibited CsA-induced HO-1 expression (Fig. 6B). These results clearly suggest that CsA-induced c-Met-HO-1 pathway plays an important role in renal tumor growth; and HNK is effective in inhibiting both CsA-induced c-Met activation and HO-1 over-expression *in vivo*.

## Discussion

The natural product HNK, which is a small molecular weight polyphenol, has been shown to have potent antitumor activity^3^. c-Met-mediated Ras activation plays a major role in the accelerated growth of renal cancer, which is also a critical problem in immuno-suppressed patients^4, 19, 21, 24^. Here, we report that HNK can effectively inhibit c-Met-mediated growth promoting pathways in RCC, involving the cytoprotective molecule HO-1. Our findings also suggest that CNI (CsA), which is used for the treatment of immune disorders, enhances c-Met activation and augments HGF/c-Met-induced Ras activation in renal cancer cells; and that HNK treatment down-regulates c-Met activation to inhibit CNI-induced renal tumor growth. Our observations show that HNK can effectively target c-Met-Ras-HO-1 pathway to restrict renal tumor growth, suggesting its use as a novel therapeutic agent in RCC.

Ras mutation is not very common in renal cancer. One of the possible mechanism(s) for hyper-activation of the Ras pathway in renal cancer cells (including the cells used in this study) is due to the signaling through upstream receptor tyrosine kinases. Our recent report suggests that HGF/c-Met-mediated signaling promotes Ras activation in renal cancer cells^4^. Here, we show that the HNK treatment can not only inhibit HGF-induced c-Met phosphorylation but also effectively suppress HGF/c-Met-mediated Ras activation. We speculate the role of HNK-induced cellular phosphatase(s) in the down-regulation of c-Met phosphorylation, based on the reports that HNK induces SHP-1, a protein tyrosine phosphatase, to down-regulate STAT3 phosphorylation; and SHP-1 can play a role in the down-regulation of c-Met phosphorylation^34, 35^. However, further studies are needed to completely understand the molecular events associated with HNK-induced down-regulation of c-Met phosphorylation.

In our previous studies, we have demonstrated that CNI treatment induces Ras activation and promotes renal cancer cell growth; and this effect of CNI is independent of its immunosuppressive function^23, 24^. Here, we found that CNI augments c-Met activation in a ligand independent manner and also enhances HGF/c-Met-induced Ras activation. However, the effector molecule(s) that mediate CNI-induced c-Met phosphorylation needs to be identified. It is known that Raf kinase inhibitory protein (RKIP) disrupts the interaction of Ras-Raf-ERK and phosphorylation of RKIP relieves the inhibitory effect of the molecule and promotes downstream signaling^36^. We have previously reported that HNK effectively inhibits CNI-induced phosphorylation of RKIP, and thereby blocks Ras downstream signaling^27^. Hence, HNK can effectively inhibit CNI-induced growth promoting signals. It has been reported that ligand independent activation of c-Met increases tumor burden and it would be of interest to know whether c-Met is activated in cancers arising in patients treated with CNI^37^. Nevertheless, our findings emphasize the potential cross-talk between CNI- and c-Met-induced tumorigenic pathways.

Previously, we have reported that both c-Met- and CNI-mediated Ras activation leads to HO-1 over-expression in renal cancer cells^4, 14, 27^. We have also demonstrated the nuclear translocation of Nrf2, the transcriptional activator of HO-1, plays critical role in promoting c-Met-mediated renal cancer cell survival^4^. In this study, we found that HNK can effectively inhibit the nuclear translocation of Nrf2 and down-regulates both c-Met- and CNI-induced HO-1 over-expression in renal cancer cells. In addition to HO-1 over-expression, c-Met-mediated increase in the expression of anti-apoptotic molecules, such as Bcl-2 and Bcl-XL, protects renal cancer cells from apoptosis. It has been reported that HNK exerts its apoptotic function on cancer cells possibly through cell type specific pathways, which includes p53 activation, inhibition of mTOR complex, caspase as well as PARP cleavage, and downregulation of Bcl-XL^38–40^. In renal cancer cells, our observation suggests that, HNK can promote cancer cell apoptosis through the down-regulation of c-Met-induced Bcl-2 and Bcl-XL expression, and through increased cleavage of caspase-3. Importantly, both c-Met hyper-activation and CNI treatment can promote metastasis of cancer cells^5, 30^; and increased tumor cell migration is the cause for metastasis^28^. Here, we also report that HNK inhibits both c-Met- and CNI-induced induced renal cancer cell migration, suggesting that HNK can have therapeutic potential in restricting metastasis of RCC.

In addition to promoting cell survival, both c-Met-mediated signaling and HO-1 can induce vascular endothelial growth factor (VEGF) expression and promote tumor angiogenesis^16, 41, 42^. CNI treatment also increases VEGF expression in renal cancer cells^32, 43^. We have previously demonstrated that HNK can inhibit Ras-mediated over-expression of VEGF in renal cancer cells^27^. It has been suggested that HNK inhibits transcription factors NF-κb and STAT3, which regulate VEGF secretion in tumor microenvironment, and HNK inhibits angiogenic signals through the downregulation of VEGF receptor 2 (VEGFR2) phosphorylation^34, 44–46^. However, the effect of HNK on renal tumor angiogenesis is not studied. In this study, we found that HNK treatment can inhibit both c-Met and CNI-induced secretion of angiogenic factors from renal cancer cells, as evident from the decrease in the extent of endothelial cell tube formation. Therefore, our findings suggest an important role of HNK to attenuate angiogenesis and vascularization that plays a crucial role in the development of renal tumors, which are highly vascular.

Although the chemotherapeutic effects of HNK on various malignancies have been reported, studies exploring the anti-tumorigenic effect of HNK on renal tumors *in vivo* are limited^47–49^. Also, there is an unmet need to identify novel therapeutic drugs/approaches to effectively suppress CNI-induced tumor growth^19^. HNK is one such promising drug with both chemopreventive and anti-inflammatory properties^1^; therefore, the use of HNK as a therapeutic for patients after organ transplantation can potentially have dual benefit, i.e. to restrict CNI-induced cancer and to mitigate the dose of CNI used to achieve immunosuppression in patients. In our renal tumor xenograft study, we found that CNI-induced increased tumor growth is associated with increased c-Met activation in tumor tissues. Importantly, HNK significantly inhibited CNI-induced tumor growth and is associated with decreased c-Met activation, which correlates with our *in vitro* findings. HNK inhibited CNI-induced HO-1 expression in tumor tissues and increased tumor cell apoptosis. We have also observed that CNI treatment increased the vessel density in tumor tissues; and it decreased with HNK combination treatment. Therefore, our *in vivo* findings suggest that HNK targets c-Met-HO-1 pathways in restricting renal tumor growth. c-Met inhibitors are being used in clinic for the treatment of RCC, and it will be interesting to further explore the potential benefits of using HNK in combination therapy^5, 50^. Through this study, we also provide the basis for the use of HNK in preventing post-transplantation cancer.

In summary, for the first time our studies demonstrate that HNK inhibits c-Met-mediated tumorigenic pathways. We found that c-Met activation and HO-1 overexpression plays a critical role in CNI-induced renal tumor growth; and it is markedly inhibited by HNK treatment. Our data clearly suggest that HNK targets c-Met-Ras-HO-1 tumorigenic pathways, and a combination therapy with HNK and other target-specific agent(s) can have promising benefits for the treatment of renal tumors, with particular importance to post-transplantation cancer.

## Materials and Methods

### Cell Lines

Human renal cancer cell lines 786-0 and ACHN (ATCC, Manassas, VA) were cultured in RPMI 1640 and EMEM medium respectively, supplemented with 10% fetal bovine serum (FBS) (Invitrogen, Carlsbad, CA). All cell lines were authenticated by Short Tandem Repeat (STR) profiling from ATCC in December, 2015. The cell lines were passaged for less than three months after thawing. Human Vascular Endothelial cells (HUVEC) were obtained from Lonza and were grown in EBM-2 basal medium supplemented with EGM-2 singlequots (growth supplements) (Lonza, USA).

### Reagents

Synthesized pure Honokiol (HNK) (C_18_H_18_O_2_, Molecular Weight: 266.334) and CsA were purchased from Sellekchem (Houston, TX) and were dissolved in DMSO. The recombinant human HGF was purchased from Peprotech (Rocky Hill, NJ).

### Western Blot Analysis

Protein samples were run on SDS polyacrylamide gel (Bio-Rad, Hercules, CA) and transferred to a polyvinylidene difluoride membrane (Millipore Corp., Billerica, MA). The membranes were incubated with anti-phospho-MET, anti-c-MET, anti-HO-1, anti-Bcl-xL, and anti-Bcl-2, anti-cleaved-caspase-3, anti-GAPDH, (Cell Signaling, Danvers, MA), anti-Nrf2, anti-Sp1 (Santa Cruz Biotechnology, Dallas, TX); or anti-β-Actin (Sigma-Aldrich, St. Louis, MO); and subsequently incubated with peroxidase-linked secondary antibody (Santa-Cruz). Reactive bands were detected using chemiluminescent substrate (Thermo Fisher Scientific, Waltham, MA). Frozen tumor tissue samples were lysed on ice with RIPA buffer; and the lysates were centrifuged and the supernatants were subsequently used for Western blot analysis. For all Western blots, 10 ug of each sample was loaded, and the primary and secondary antibodies were diluted according to the manufacturer’s recommendation.

### Measurement of Active/GTP bound Ras

The active/GTP-bound form of Ras in the cell lysates was measured using an EZ-detect Ras activation kit (Thermo Fisher Scientific, Waltham, MA).

### Cell Proliferation Assay

MTT cell proliferation assay (ATCC, Manassas, VA) kit was used to quantify cell proliferation, following manufacturer’s protocol.

### Apoptosis Assay

Cellular apoptosis was measured using an allophycocyanin (APC)-conjugated Annexin-V and propidium iodide (PI) apoptosis detection kit (Thermo Fisher Scientific, Waltham, MA). Following staining, the cells were analyzed by flow cytometry on a FACSCalibur.

### Preparation of Nuclear Extracts

Nuclear extracts were prepared from the cells using a nuclear extraction kit (Active Motif, Carlsbad, CA), following manufacturer’s protocol.

### *In vitro* wound healing assay

Cells were seeded on 6-well culture plates. When cells were grown to confluence, medium was aspirated and, using a 200 μL pipette tip, cells were scratched along the diameter of the well, to simulate the wound. Cells were washed twice with PBS and were treated in serum-free medium as indicated. At 0 h and 12 h after incubation, wound diameter in each well was photographed using phase contrast microscope to assess wound closure. Number of cells migrated to the wound area beyond the reference line were quantified using NIH ImageJ software.

### Endothelial cell tube formation assay

Conditioned medium from cancer cells was collected and utilized to culture HUVECs (2 × 10^4^ cells) on a layer of previously polymerized growth factor-reduced extracellular matrix (Geltrex)(Invitrogen, Carlsbad, CA) in 6-well culture plates. After 6 h, HUVEC culture well was photographed through a phase contrast microscope to assess changes in HUVEC morphology and capillary-like tube formation. The extent of tube formation was quantified by counting the number of uninhibited polygonal tube-like structures in five randomly selected microscopic fields.

### *In vivo* tumor development in a Xenograft model

Animal experiments were performed following all guidelines in accordance with Institutional Animal Care and Use Committee (IACUC) protocol approved by Boston Children’s Hospital. 786-0 cells (1 × 10^6^ cells/mice) were injected subcutaneously into the flanks of six-week old immunodeficient (nude) mice (NU/J, Jackson Laboratory, Bar Harbor, ME). Once palpable tumors were formed, mice were randomly distributed to different treatment groups as described. CsA and HNK were diluted in normal saline to reach the desired concentration before injection. Tumor volume was measured using digital caliper measurements obtained at regular intervals. The volume was estimated by following standard method, using the formula *V* = *π*/*6* × *a* ^*2*^ × *b* where *a* is the short axis and *b* is the long tumor axis. At the end of the study, tumors were excised and processed for further studies.

### Immunohistochemistry

Tumor xenografts were excised and rapidly frozen in OCT compound (Tissue-Tek, Torrance, CA) and tumor tissue sections were cut on a cryostat (Leica, Buffalo Grove, IL). Following standard protocols, the sections were processed for immunolabeling with anti-CD31 (1:50 dilution) (Abcam, Cambridge, MA), anti-phospho-c-Met (1:300 dilution), total-Met (1:300 dilution) (Cell Signaling, Danvers, MA) or anti-HO-1 (1:50 dilution) (Novus Biologicals, Littleton, CO); and subsequently labelled with a species-specific horseradish peroxidase-conjugateed secondary antibody. In tissue sections stained with anti-CD31 antibody, mean vessel density was calculated by standard grid counting method at x400 magnification.

### Statistical analysis

Changes in mean tumor volume were compared by two-way repeated-measures analysis of variance (ANOVA) to test for differences in slopes between the treatment groups over time using the group-by-time interaction F-test. Fit to the data in handling tumor volume measurements from the same animal at the serial time points was accounted for using a compound symmetry covariance structure in the mixed-model longitudinal approach and this provided excellent fit to the data, as judged by the Akaike information criterion (AIC). Data are presented as mean and standard deviation (SD). Power analysis indicated that sample sizes of *n* = 5 mice per group will provide 80% statistical power (2-tailed α = 0.05, β = 0.20) to detect significant differences of 50% or more in tumor volume between the groups. Statistical analysis was performed using SPSS software version 23 (IBM Corporation, Armonk, NY). Conservative two-tailed values of p < 0.01 were considered statistically significant in order to protect against type I errors due to multiple group comparisons. For *in vitro* experiments, statistical significance was determined by Student’s *t* test. Differences with *p* < 0.05 were considered statistically significant.

## Electronic supplementary material

Supplementary information
