# Supplementary material for: Honokiol inhibits c-Met-HO-1 tumor-promoting pathway and its cross-talk with calcineurin inhibitor-mediated renal cancer growth
Source: Sci Rep. 2017 Jul 19;7:5900. doi: 10.1038/s41598-017-05455-1 (PMC5517643; doi:10.1038/s41598-017-05455-1)
Supplement: Supplementary file 1 — Supplementary information [file 41598_2017_5455_MOESM1_ESM.pdf]

Supplementary file

Title:

Honokiol inhibits c-Met-HO-1 tumor-promoting pathway and its cross-talk with calcineurin inhibitor-mediated renal cancer growth

Authors:

Murugabaskar Balan[a,c], Samik Chakraborty[a,c], Evelyn Flynn[a,c], David Zurakowski[b,c] and Soumitro Pal[a,c,]*

[a]Division of Nephrology, Boston Children's Hospital, MA 02115; [b]Department of Anesthesia, Boston Children's Hospital, MA 02115; and [c]Harvard Medical School, Boston, MA 02115;

Figure-1A

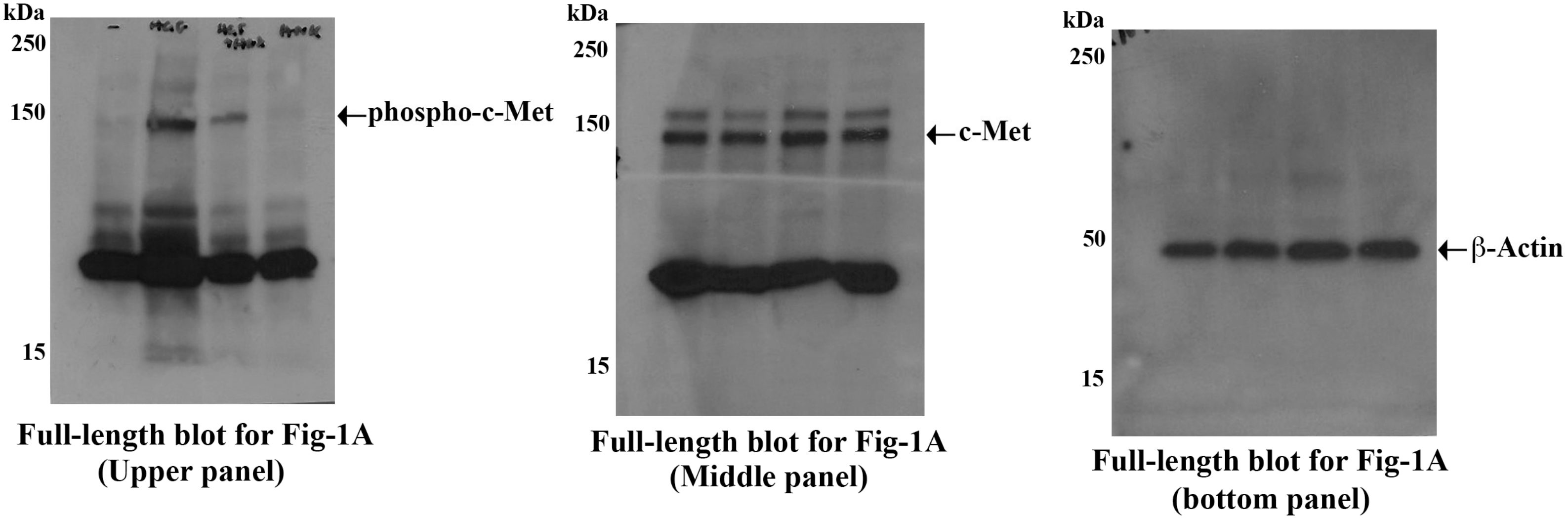

Full-length blot for Fig-1A (Upper panel)

Full-length blot for Fig-1A (Middle panel)

Full-length blot for Fig-1A (bottom panel)

Figure-1B

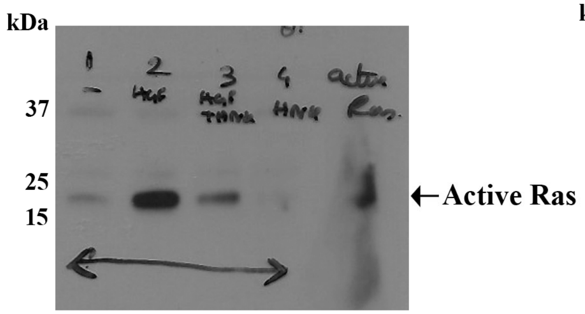

Full-length blot for Fig-1B (upper panel)

Full-length blot for Fig-1B (lower panel)

Figure-1C

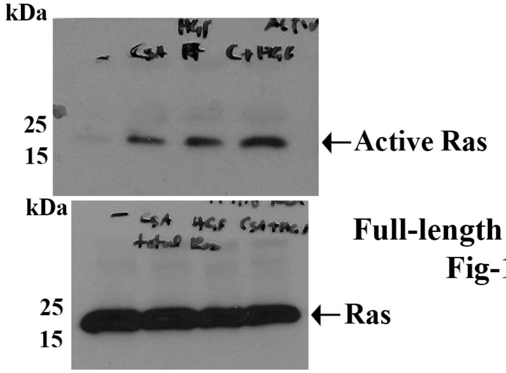

Full-length blots for Fig-1C

Figure-1D

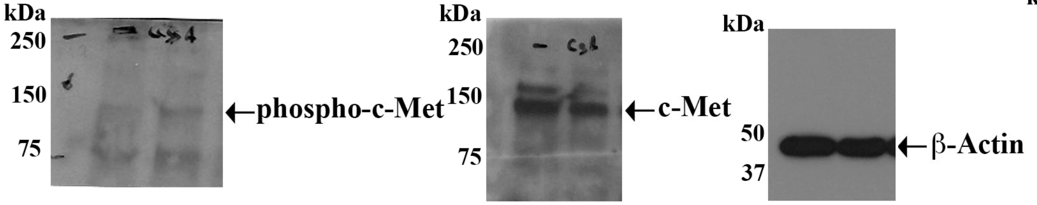

Full-length blots for Fig-1D

Figure-1E

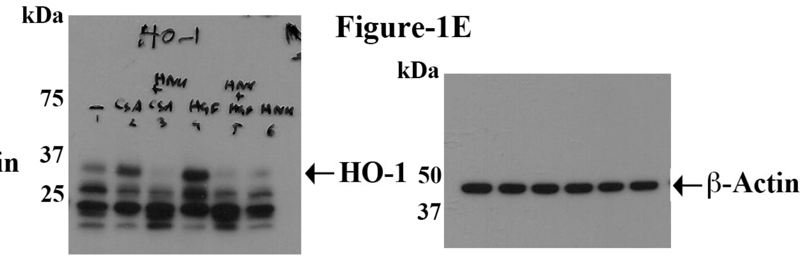

Full-length blots for Fig-1E

Figure-1F

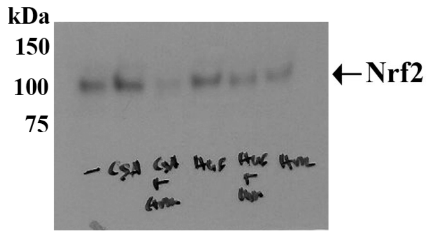

Full-length blot for nuclear Nrf2 in Fig-1F

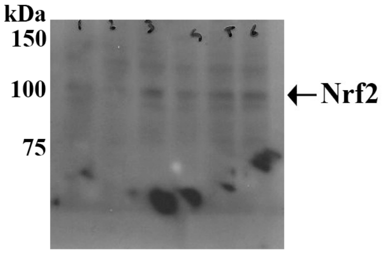

Full-length blot for cytoplasmic Nrf2 in Fig-1F

Figure-2C

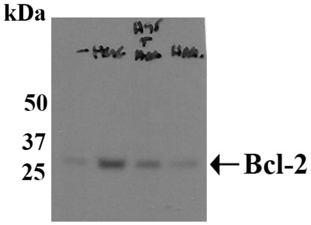

Full-length blot for Fig-2C (upper panel)

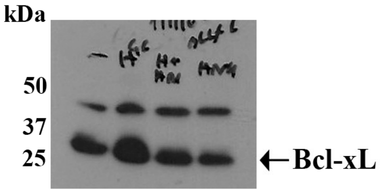

Full-length blot for Fig-2C (middle panel)

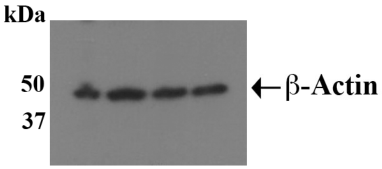

Full-length blot for Fig-2C (lower panel)

Figure-5D

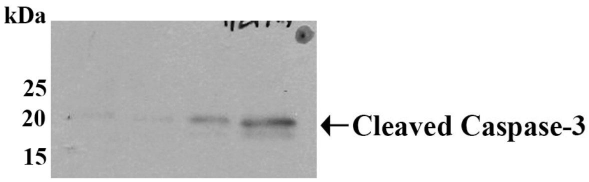

Full-length blot for Fig-5D (upper panel)

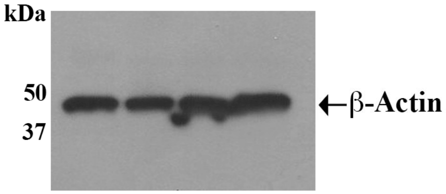

Full-length blot for Fig-5D (lower panel)
